# Effect of Vulcanization and CO_2_ Plasticization on Cell Morphology of Silicone Rubber in Temperature Rise Foaming Process

**DOI:** 10.3390/polym13193384

**Published:** 2021-10-01

**Authors:** Tianping Zhang, Shun Yao, Lu Wang, Weijun Zhen, Ling Zhao

**Affiliations:** 1 School of Chemical Engineering and Technology, Xinjiang University, Urumqi 830046, China; zhangtping@163.com (T.Z.); wanglu_4951@163.com (L.W.); zhenweijun6900@163.com (W.Z.); 2 Shanghai Key Laboratory of Multiphase Materials Chemical Engineering, East China University of Science and Technology, Shanghai 200237, China; yaoshun0727@163.com

**Keywords:** methyl-vinyl silicone rubber, temperature rise foaming process, vulcanization reaction, CO_2_ plasticization, rheological property, cell morphology

## Abstract

Both vulcanization reaction and CO_2_ plasticization play key roles in the temperature rise foaming process of silicone rubber. The chosen methyl-vinyl silicone rubber system with a pre-vulcanization degree of 36% had proper crosslinked networks, which not only could ensure enough polymer matrix strength to avoid bubble rupture but also had enough dissolved CO_2_ content in silicone rubber for induced bubble nucleation. The CO_2_ diffusion and further vulcanization reaction occur simultaneously in the CO_2_ plasticized polymer during bubble nucleation and growth. The dissolved CO_2_ in the pre-vulcanized silicone rubber caused a temperature delay to start while accelerating further vulcanization reactions, but the lower viscoelasticity caused by either CO_2_ plasticization or fewer crosslinking networks was still the dominating factor for larger cell formation. There was a sudden increase in elastic modulus and complex viscosity for pre-vulcanized silicone rubbers at higher temperature because of the occurrence of further vulcanization, but CO_2_ plasticization reduced the scope of change of rheological properties, and the loss factor was close to 1 around 170 °C, which is corresponding to the optimum foaming temperature. The foamed silicone rubber had a higher cell density and smaller cell size at a higher temperature rising rate, which is due to higher CO_2_ supersaturation and faster vulcanization reaction. These results provide some insight into the coupling mode and effect of CO_2_ plasticization and vulcanization for regulating cell structure in foaming silicone rubber process.

## 1. Introduction

Silicone rubbers have excellent wide temperature range suitability, favorable weather resistance and chemical resistance, good dielectric properties and physiological inertia, and high gas permeability due to their unique inorganic–organic hybrid structure. Silicone rubber foam materials further have interesting properties such as damping, lightweight, heat insulation, and sound absorption [1,2], and they are widely used in transportation, aerospace, electronic equipment, telecommunications, biomedicine, packaging, and home construction as elastic and soft materials. Apart from the well-known hydrosilylation/condensation foaming technology [3,4], cellular silicone rubbers can also be produced by using conventional chemical blowing agents, particle leaching, phase separation, templated foaming, 3D printing, and gas foaming. In recent years, especially many researchers are moving toward the supercritical carbon dioxide (CO_2_) foaming process due to its environmentally friendly nature and superior capability for producing a microcellular structure. Silicones have also good CO_2_ compatibility [5]. However, the CO_2_ physical foaming of silicone rubber can generate microsized and even nanosized cells and a high cell density, which can result in high gas solubility and diffusivity for effective foaming. Generally, chemical foaming of silicone rubber, because of H_2_ production, can result in low foam density but with big cells [6].

Though supercritical CO_2_ is a physical blowing agent, it is inevitable for silicone rubber to undergo a vulcanization reaction during the CO_2_ foaming process. Since the crosslinking networks control the chain mobility and rheological properties of silicone rubber, the progress of the vulcanization reaction should have a proper match with the foaming process. It is difficult to simultaneously control the vulcanizing and CO_2_ foaming process, so the strategy of separating foaming and vulcanizing has been performed, that is, partially crosslinked silicone rubber is firstly obtained by controlling different vulcanization conditions; then, this pre-vulcanized sample is saturated under high-pressure CO_2_, the bubble nucleation happens via fast depressurization or temperature rising afterward, and finally, the foamed sample is post-vulcanized to stabilize cell morphology and enhance mechanical properties. Shimbo et al. [7] obtained a foamed silicone rubber with a cell size of 100 μm by adjusting the vulcanization degree. They found that the vulcanization degree of silicone rubber before foaming was crucial to achieving the desired cell morphology. Insufficient vulcanization would lead to unconstrained cell growth and cell coalescence, and the rigid crosslinking networks at a high level of vulcanization would hinder bubble nucleation and growth. Hong and Lee [8] also disclosed that there existed a proper cure degree for a well-defined cell structure after investigating the rheological behavior during the crosslinking of silicone rubber. The vulcanization time was an important parameter to control the cell morphology of the crosslinked silicone rubber [9]. Liao et al. [10] found that the silicone rubber can be foamed at a CO_2_ saturation pressure of 10–14 MPa with a longer pre-curing time of 18 min, whereas for the silicone rubber sample with a shorter pre-curing time of 6 min, a uniform and well-defined cellular structure could be fabricated under 10 MPa, because the cell structure became unstable due to the lower viscosity and lower modulus under higher pressures of 12 MPa and 14 MPa. Xu et al. [11] found that increasing the pre-curing time in a short time had a greater effect on cell nucleation than cell growth during foaming. Jia et al. [12] discovered that the plasticity of the silicone rubber matrix plays an important role in cellular formation. The degree of retraction increased during cell fixation when the proportion of elasticity in the silicone rubber matrix increased with a longer pre-curing time.

There is a strong interaction between CO_2_ and silicone rubber [13], which can lead to a strong CO_2_ plasticization effect. Yang et al. [14] found that the CO_2_ concentration of around 3% and diffusivity with a magnitude of 10^−5^ cm^2^/s in high temperature vulcanized methyl vinyl silicone rubber at a CO_2_ pressure of 2–5 MPa at 40 °C, and this diffusion coefficient of CO_2_ in silicone rubber was 1000 times higher than that in polyetherimide. Yan et al. [15] fitted the cell density with CO_2_ dissolution and found that the logarithm of the cell density had a linear relationship with the square of saturation pressure. Liao et al. [10] characterized viscoelasticity thoroughly based on rheology measurements with and without CO_2_. Their results measured by a high-pressure rotational rheometer showed that both the complex viscosity and storage modulus decreased due to the more CO_2_ permeation into the silicone rubber under higher CO_2_ saturation pressure, but the effect of the CO_2_ saturation temperature on the viscoelastic properties of silicone rubber is much more complex because the curing agent’s decomposition and the CO_2_ plasticization have two opposite effects. The in situ high-pressure rheological test during CO_2_ saturation in the work of Tang et al. [16] also showed that the storage modulus and complex viscosity gradually decreased with time to an equilibrium until the CO_2_ saturated in the silicone rubber matrix, and the complex viscosity decreased faster and more at lower crosslinking density.

Some studies also focused on different formula compositions as well as functional fillers in silicone rubbers for good cell structure and to improve the mechanical properties of the obtained foams. Yan et al. [15] studied high-temperature vulcanized silicone rubber foam material and found that increasing the content of silica could reduce the cell size of silicone rubber bubbles. Bai et al. [17] used supercritical CO_2_ to foam poly-methyl-vinyl siloxane and found that the introduction of nanometer-sized graphene into polymer matrix as a nucleating agent could reduce the size of foam cells and improve the mechanical strength of silicone rubber foam material. Messinger et al. [18] studied the influence of the microscopic properties such as molecular composition, molecular structure, intermolecular interaction, and foam structure on the macroscopic mechanical properties of the silicone foam material. They found that silicone foam materials were stronger with a higher crosslinking degree and fewer side chain phenyl groups. Liu et al. [19] found that polyhedral oligomeric silsesquioxane (POSS) particles with grafted carboxylic acid groups could improve the strength of silicone rubber foam, but POSS grafted with carboxylic acid groups played an inhibitory role in the curing process. Shi et al. [20] prepared silicon rubber/functionalized graphene nanocomposite foam with reinforced mechanical properties, and the uniformly dispersed 3-aminopropyltriethoxysilane functionalized graphene significantly enhanced the matrix strength, which was beneficial for limiting the shrinkage of the cell wall.

Although the effect of pre-vulcanization of silicone rubbers on their CO_2_ foaming behavior has been investigated a lot, few studies involve the further vulcanization reaction during bubble nucleation and growth, and the effect of CO_2_ plasticization on the vulcanization reaction has been also rarely concerned until now. The effect of CO_2_ plasticization on polymer matrix strength has been known from the rheological properties of silicone rubber under a high CO_2_ pressure atmosphere, but the rheological behavior of CO_2_ plasticized silicone rubbers may change with the dissolved CO_2_ diffusion out of a polymer matrix continuously during bubble formation. In this work, the effect of complex chemical and physical changes on cell morphology will be studied in the temperature rise foaming process of silicone rubber using supercritical CO_2_ as a blowing agent. The samples with different pre-vulcanization degrees first are prepared and foamed under optimum conditions, and the proper pre-vulcanization degree with good cell morphology can be chosen. Then, the rheological and non-isothermal DSC tests will be carried out for the pre-vulcanized samples after being saturated under different CO_2_ pressure and time, which can be helpful to deeply understand the effect of CO_2_ plasticization on cell morphology. Finally, combined with the rheological properties and further vulcanization degree rising in different temperature change courses, the influence of both vulcanization degree and rate on bubble nucleation and growth is explored.

## 2. Experimental Section

### 2.1. Materials

The methyl-vinyl silicone rubber raw gum had a molecular weight of 600,000 g/mol. The hydroxyl silicone oil contained 6.0–12.0% hydroxyl group, and the viscosity was less than 20 mm^2^/s at 35 °C. Silica powder (HS−200) had the specific surface area of 185–225 m^2^/g. All the above materials were provided by HeSheng Silicon Industry Co. Ltd. (Jiaxing, Zhejiang, China). Dicumyl peroxide (DCP, purity > 99%) was purchased from Shanghai Aladdin biochemical technology Co. Ltd. (Shanghai, China). Silica powder was pretreated for 180 min at 75 °C in an oven to remove the moisture and have better dispersion.

### 2.2. Pre-Vulcanization Process

All components, including methyl vinyl silicone rubber 100 phr, filler silica in 25 phr, hydroxyl silicone oil in 4 phr, and dicumyl peroxide in 1 phr, were well mixed in a micro extruder (HAAKE Mini Lab II, Thermo Fisher Scientific, Waltham, MA, USA) at room temperature to obtain silicone rubber sample; then, this sample was placed at 150 °C in an oven to obtain pre-vulcanized samples with different vulcanization degrees by controlling time.

### 2.3. Step Temperature-Rising Foaming Process

**Figure 1:**
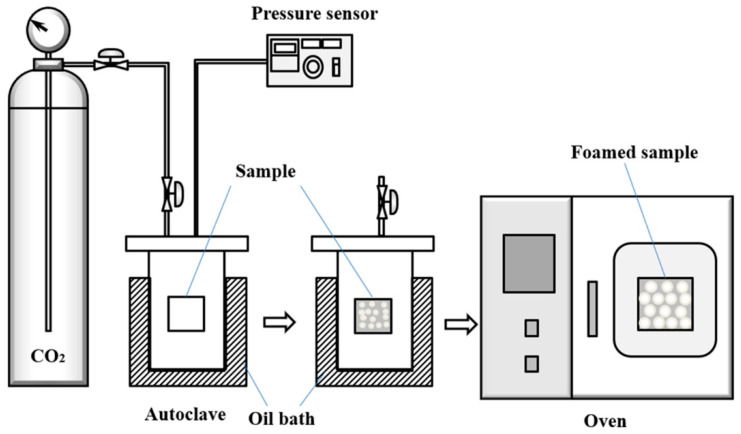
Diagram of CO_2_ foaming silicone rubber devices.

Figure 1 is the diagram of a foaming device. The pre-vulcanized silicone rubber sheet sample with 0.5 mm thickness was put into an autoclave of 250 mL, and the air in this autoclave was replaced by slowly purging with low-pressure CO_2_ for three times. Then, the autoclave was placed in an oil bath at 35 °C, and CO_2_ at different pressure was introduced into silicone rubber for 30–180 min before slow depressurization. After pressure relieving, the sample was quickly transferred into an oil bath at 150–180 °C to conduct a rising temperature foaming stage. Finally, the foamed sample was kept in the oven at 200 °C for 120 min for a complete post-vulcanization.

The CO_2_ saturation in preparation of CO_2_ plasticized samples was the same as the foaming process. After CO_2_ saturation, the pressure was released within 90 s, and silicone rubber sheet samples were taken out for further measurements. The CO_2_ content dissolved in silicone rubber was determined by the desorption method [21]. The CO_2_-saturated samples were quickly put on the balance to record its weight change over time at atmospheric pressure. The desorption curve was extrapolated to obtain CO_2_ concentration based on polymer.

### 2.4. Characterization

#### 2.4.1. Differential Scanning Calorimetry (DSC) Analysis

The vulcanization degree of different silicone rubber samples including after pre-vulcanization, CO_2_ saturation, and foaming was determined by differential scanning calorimetry (DSC, TADHR-2, TA Instrument Inc., New Castle, DE, USA) at a heating rate of 5 °C/min. The vulcanization degree value was calculated by Equation (1).

(1) $$\alpha =1-\Delta H_{t}/\Delta H_{\mathrm{total}}$$

where *α* is the vulcanization degree of the sample, Δ*H_t_* is the vulcanization reaction exothermic enthalpy of vulcanized samples, and Δ*H_total_* is the vulcanization reaction exothermic enthalpy of the un-vulcanized sample. The reaction exothermic enthalpies were calculated by the DSC curve.

#### 2.4.2. Rheological Properties Measurements

The rheological properties of mixed rubber samples were characterized by a rotational rheometer (TA DHR-2, TA Instrument Inc., New Castle, DE, USA) with a 25 mm diameter plate under atmospheric pressure. The thickness of the samples was 1 mm. Both pre-vulcanized silicone rubber samples and CO_2_ plasticized silicone rubber samples were subjected to an oscillation shear test at 35 °C, the oscillation frequency was 0.1–100 rad/s, and the strain was 1%. Dynamic temperature scanning of CO_2_ plasticized silicone rubber samples was carried out under atmospheric pressure. The fixed frequency was 1 Hz and 1% for the control strain, and the temperature scanning range was 30–250 °C.

#### 2.4.3. Foam Characterization

The density of silicone rubber samples before and after foaming was measured by the drainage method. The following formula is the calculation method.

(2) $$\rho =\left[m_{0}/\left(m_{2}+m_{0}-m_{1}\right)\right]\rho_{water}$$

$m_{0}$ is the mass of the sample in the air, $m_{1}$ is the mass of the sample in a density bottle filled with water, $m_{2}$ is the mass of the density bottle full of water, and $\rho_{water}$ is the density of water [22].

The cell morphology of the silicone rubber foam sample was characterized by scanning electron microscope (SEM, 1430VP, Carl Zeiss AG, Jena, Germany). The average cell size was analyzed by the Image-Pro Plus software, and the following formula was used to calculate the silicone rubber cell density *(N_f_*) [6].

(3) $$N_{f}={\left(n/A\right)}^{3/2}\left(\rho_{0}/\rho_{f}\right)$$

When *n* is the number of cells in the microscope photograph, *A* is the area of the micrograph. $\rho_{\begin{array}{c} 0 \end{array}}$ is the density of silicone rubber before foaming (1.06 g/m^3^), and $\rho_{f}$ is the density of fully vulcanized silicone rubber after foaming.

## 3. Results and Discussion

### 3.1. Foamable Range of Pre-Vulcanization Degree

**Figure 2:**
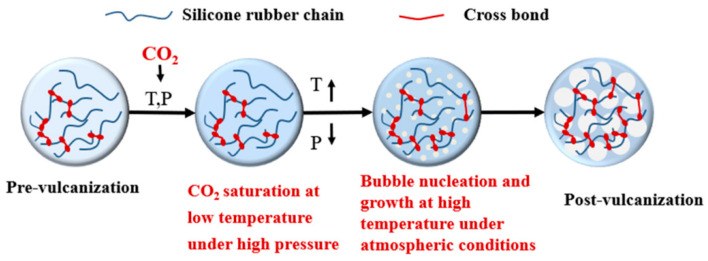
Schematic diagram of foaming silicone rubber process.

There are four stages of pre-vulcanization, CO_2_ saturation, bubble nucleation and growth, and post-vulcanization in the temperature rise foaming process, as shown in Figure 2. The pre-vulcanization degree can be controlled by the reaction temperature and time. The temperature during CO_2_ saturation is controlled at low temperature so that the vulcanization reaction does not further happen. The CO_2_ content in the pre-vulcanized silicone rubber sample can be adjusted by CO_2_ saturation pressure and time. The CO_2_ diffusion and further vulcanization reaction occur simultaneously in CO_2_ plasticized polymer during bubble nucleation and growth, since the bubble nucleation happens because of the thermodynamic instability induced by the rising temperature, and the bubbles continue to grow up at a certain higher temperature for a period of time. Final post-vulcanization at high temperature aims to achieve complete crosslink networks in foamed samples for stable cell morphology. Therefore, the vulcanization degree of silicone rubber continues to increase except at the high-pressure CO_2_ saturation stage.

**Figure 3:**
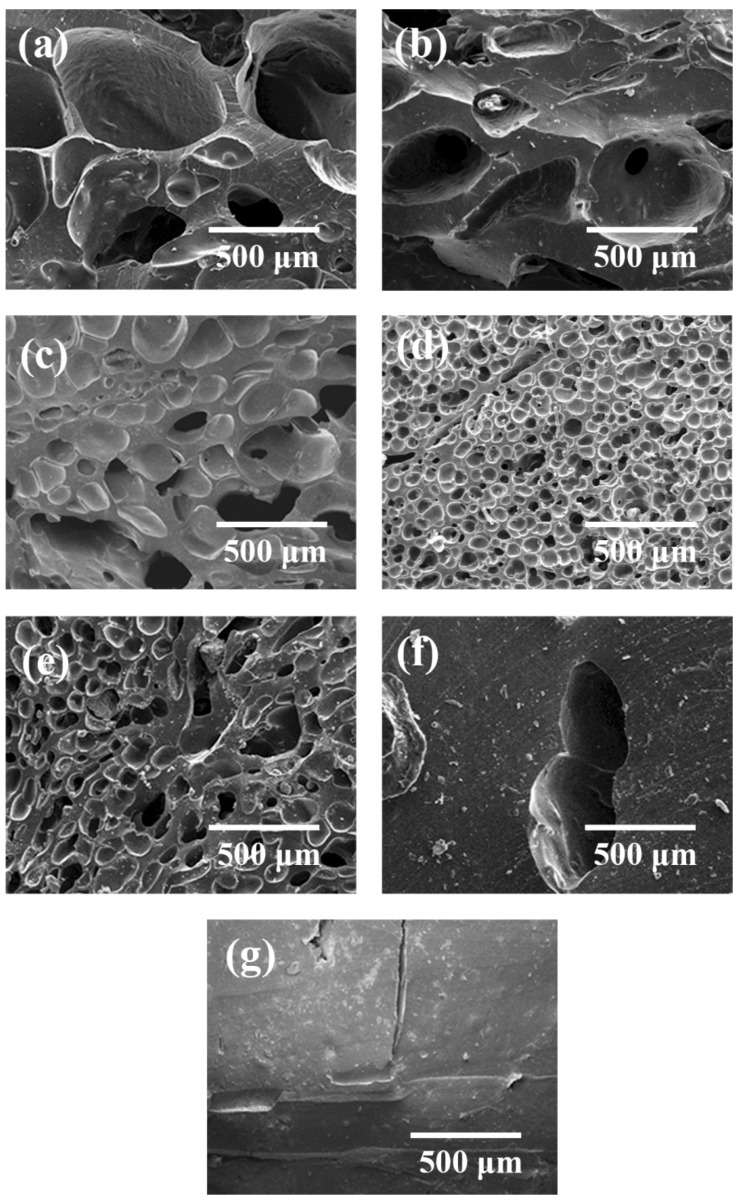
Cell morphology of foamed samples with different pre-vulcanization times: (**a**) 0 min; (**b**) 10 min; (**c**) 20 min; (**d**) 30 min; (**e**) 40 min; (**f**) 50 min; (**g**) 60 min.

Silicone rubber samples with different pre-vulcanization times were saturated under CO_2_ pressure of 10 MPa for 120 min at 35 °C and then heated to 170 °C for 60 min of foaming. The cell morphology of foamed samples is shown in Figure 3 and Table 1. When the pre-vulcanization time increased from 10 to 20 min, the vulcanization degree increased from 12.7% to 23.9%. The cell size decreased, but there was still cell rupture. The vulcanization time range varied from 30 to 40 min, and the vulcanization degree increased from 36.0% to 43.8%; meanwhile, the cell size became small, and the cell density increased. However, only a small amount of cells existed when the vulcanization was performed for 50 min. When the vulcanization time rose to 60 min, the vulcanization degree reached 56.6%, and the crosslinking process was too high to form a bubble cell structure. So, there was a pre-vulcanization degree range of 23.9−43.8% for the chosen silicone rubber system, in which the foamed samples had a smaller cell size, just as other crosslinked polymers, for example epoxy resin [23]. In the pre-vulcanized silicone rubber, there are vulcanized regions and un-vulcanized regions. The un-vulcanized part generally shows good CO_2_-dissolving capacity, which is conducive to bubble nucleation and growth [12,24], and the vulcanized regions have good viscoelasticity, which enables silicone rubber to have enough polymer matrix strength to support bubble growth without cell coalescence or cell collapse. However, many more crosslinked networks in silicone rubber not only would restrict the growth of cells but also would hinder CO_2_ dissolution.

**Figure 4:**
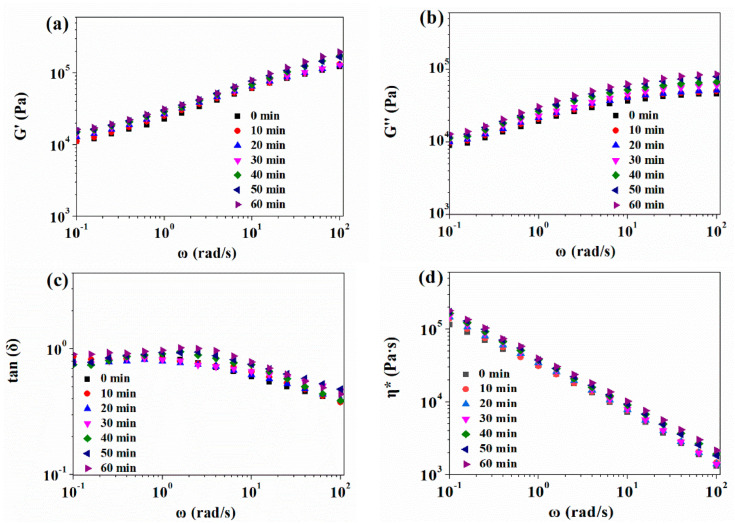
Rheological behavior of silicone rubber samples at different pre-vulcanization times at 35 °C: (**a**) Storage modulus *G′*; (**b**) Loss modulus *G″*; (**c**) Loss angle tan (*δ*); (**d**) Complex viscosity η*.

Figure 4 shows the complex viscosity (η*) as a function of frequency (*ω*) ranging from 0.1 to 100 rad/s for silicone rubber samples with different pre-vulcanization degrees; the complex viscosities change linearly with frequency, but it also could be seen that the change range of the rheological properties of these pre-vulcanized samples was not significant.

The data of CO_2_ solubility in pre-vulcanized samples are listed in Table 1, since the dense crosslinked networks in the samples with a high pre-vulcanization degree limited CO_2_ diffusion into the polymer seriously, the CO_2_ content is too low to provide sufficient nucleation driving force, only a few holes are observed, and most regions are non-foamed in Figure 3f,g. Therefore, the low CO_2_ content in samples with a pre-vulcanization degree over 50% should be the dominating factor for little bubble formation. Here, one thing needs to be noted: when the pre-vulcanization degree is lower than 20%, small molecules in the composition of silicone rubber are easily drained off by CO_2_, and the CO_2_ content in the polymer matrix cannot be determined reasonably. The samples with a pre-vulcanization degree of 36.0%, which has better cell morphology, were chosen to carry out further studies.

### 3.2. Effect of CO_2_ Plasticization Derived from CO_2_ Saturation

#### 3.2.1. Effect of CO_2_ Plasticization on Rheological Behavior

**Figure 5:**
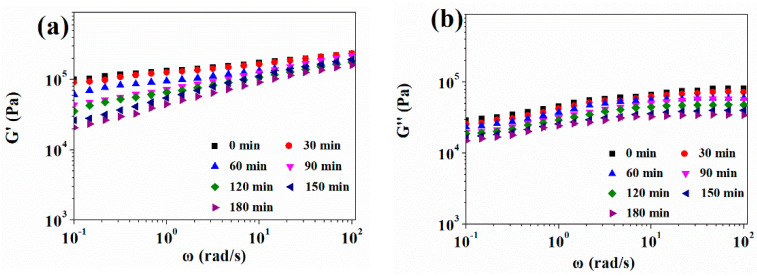

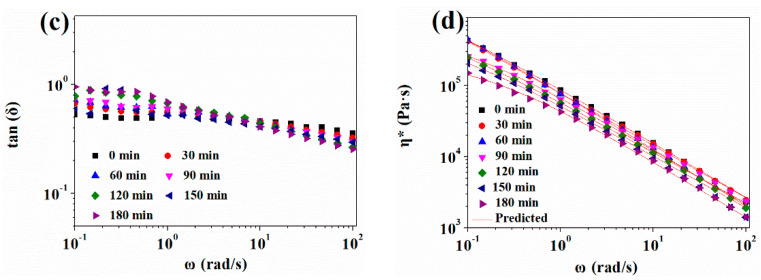
Rheological behavior of pre-vulcanized silicone rubber samples being treated with different CO_2_ saturation times at 35 °C: (**a**) Storage modulus *G′*; (**b**) Loss modulus *G″*; (**c**) Loss angle tan (*δ*); (**d**) Complex viscosity η*.

The oscillatory shear test was performed to study the rheological properties of pre-vulcanized silicone rubber after being saturated with 10 MPa CO_2_ for 30–180 min at 35 °C. As shown in Figure 5, the storage modulus, loss modulus, and complex viscosity all decreased with CO_2_ treatment time. The shear flow deformation of the silicone rubber was further determined according to Equation (4) [25], and the data-fitting results are summarized in Table 2.

(4) $$\eta^{*}=\eta_{0}/\left[1+{\left(\lambda \omega \right)}^{c}\right]$$

where η* represents the complex viscosity, *η_0_* represents the zero-shear viscosity, *λ* represents the characteristic relaxation time, *ω* represents the angular frequency, and *c* represents the Cross index.

With the increase in CO_2_ saturation time, more CO_2_ entered into the silicone rubber matrix, the free volume increased, the viscosity decreased, and the characteristic relaxation time of the chain segments motion also decreased.

#### 3.2.2. Effect of CO_2_ Plasticization on Vulcanization Reaction

The pre-vulcanization samples were saturated in different CO_2_ pressure for 120 min at 35 °C, and then, the non-isothermal DSC test was performed immediately with a 5 °C/min temperature rising rate after the CO_2_ pressure was released slowly, which corresponded to the cell nucleation and growth stage, in which CO_2_ diffusion and vulcanization reactions were carried out simultaneously. The relative conversion *α*(*t*) of the further vulcanization reaction was obtained according to Equation (5).

(5) $$\alpha \left(t\right)=\Delta H_{t}/\Delta H_{total}$$

△*H_t_* is the exothermic enthalpy of reaction at time *t*, and △*H_total_* is the total exothermic enthalpy of the reaction.

**Figure 6:**
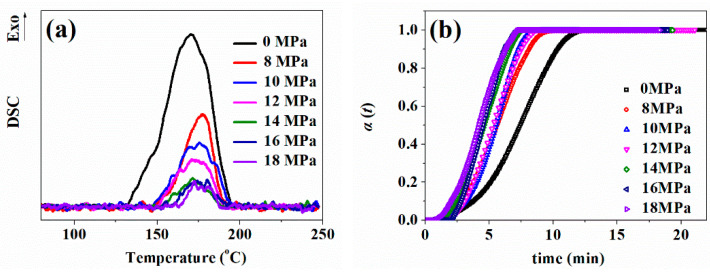
(**a**) Non-isothermal DSC curves; (**b**) Relationship between the relative conversion and the vulcanization time for pre-vulcanized silicone rubber samples after being saturated under different pressures.

Figure 6a shows non-isothermal DSC curves; dissolved CO_2_ in silicone rubber caused a delay in the starting temperature of the further vulcanization reaction. As the saturation pressure of CO_2_ increased, the content of CO_2_ increased, and the faster and more CO_2_ that escaped from polymer matrix, the more heat CO_2_ absorbed in this process, which would lead to a decrease in the exothermic peak of the vulcanization reaction. Figure 6b shows curves of the relative conversion of the further vulcanization reaction changing with time for CO_2_-plasticized silicon rubber samples. Owing to both the free volume in the polymer matrix increasing and viscosity decreasing with CO_2_ concentration, the mass transfer is enhanced, so that the vulcanization reaction rate was accelerated and the vulcanization time was shortened; this phenomenon is similar to the faster curing rate for polyurethane and epoxy resin under high CO_2_ atmosphere [26,27,28]. However, after CO_2_ pressure reached 14 MPa, the vulcanization reaction rate varied insignificantly, since the free volume of the polymer was also extruded by system static pressure [29].

#### 3.2.3. Effect of CO_2_ Saturation Time on Cell Morphology

**Figure 7:**
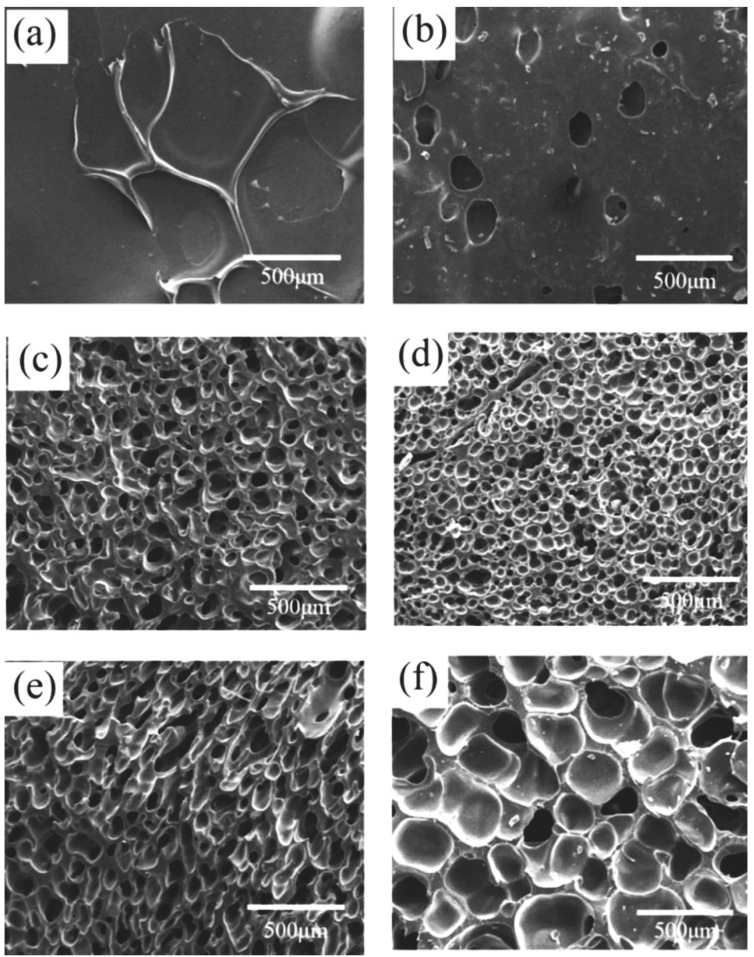
Cell morphology of foamed samples with different CO_2_ saturation times: (**a**) 30 min; (**b**) 60 min; (**c**) 90 min; (**d**) 120 min; (**e**) 150 min; (**f**) 180 min.

The pre-vulcanized samples were saturated by 10 MPa CO_2_ pressure for 30–180 min at 35 °C; then, they reached 170 °C by a heating rate of 5 °C/min and foamed for 60 min at 170 °C. The cell morphology of the foamed samples is presented in Figure 7 and Table 3. When silicone rubber samples were saturated in CO_2_ for 30 min, the solubility of CO_2_ was only 0.010 g CO_2_/g SR because of inadequate saturation time. The low solubility cannot induce the cell nucleation and form bubbles, which is illustrated in Figure 7a. With the sample being saturated for 60 min, the concentration of CO_2_ reached 0.025 g CO_2_/g SR, and only a small number of closed spherical bubbles appeared. When the CO_2_ saturation time varied from 90 to 120 min, the amount of CO_2_ that dissolved into the polymer matrix increased from 0.058 to 0.070 g CO_2_/g SR, and relatively uniform and close cells could be obtained. When the saturation time was further extended, the CO_2_ concentration increased a little and approached the CO_2_ solubility limit, but the polymer samples have been well plasticized by the higher content of dissolved CO_2_, and the polymer matrix strength became lower because of the CO_2_ plasticization effect on the rheological properties, as shown in Figure 5; therefore, bubbles began to rupture and merge, resulting in the formation of big cells.

#### 3.2.4. Effect of CO_2_ Saturation Pressure on Cell Morphology

**Figure 8:**
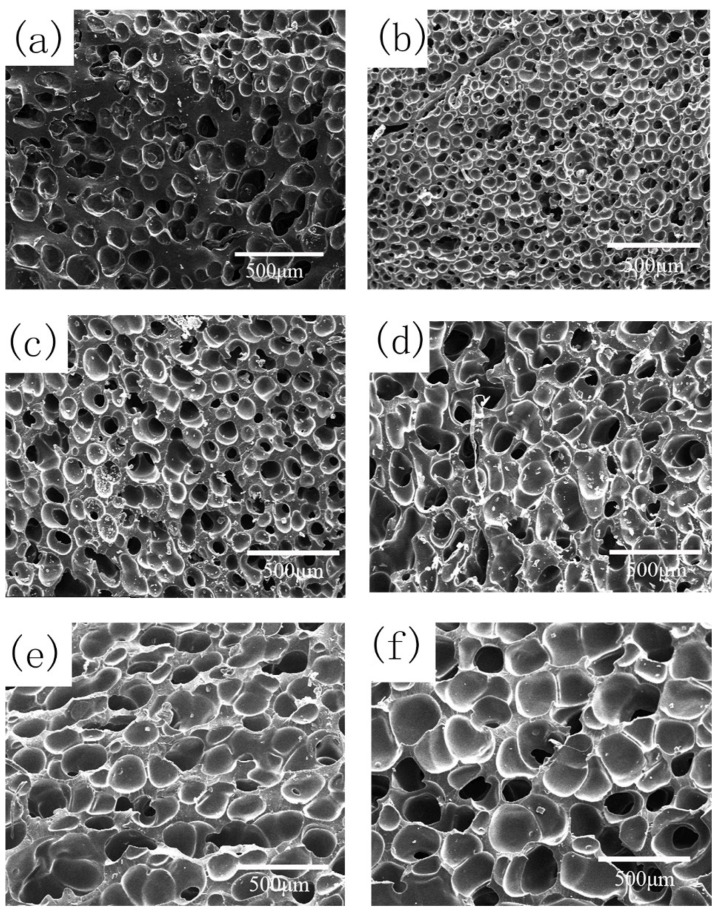
Cell morphology of foamed samples under different CO_2_ saturation pressures: (**a**) 8 MPa; (**b**) 10 MPa; (**c**) 12 MPa; (**d**) 14 MPa; (**e**) 16 MPa; (**f**) 18 MPa.

The samples were saturated under 8–18 MPa CO_2_ pressure for 120 min at 35 °C and then reached 170 °C by a heating rate of 5 °C/min and foamed for 60 min at 170 °C. The cell morphology of the foamed samples is presented in Figure 8 and Table 4. When the saturation pressure was 10 MPa, the cell density of 72.1 × 10^5^ cells/cm^3^ was the largest and the average cell diameter of 51.6 μm was the smallest. Due to the good affinity between CO_2_ and silicone rubber [30,31], the higher CO_2_ saturation pressure, and the higher dissolved CO_2_ content in the silicone rubber matrix, more bubble nucleation should happen according to the classical nucleation theory, but the cell density began to decrease at higher CO_2_ saturation pressure. This may be due to the lower polymer matrix strength caused by strong CO_2_ plasticization. On the other hand, the delayed start of the further vulcanization reaction may also result in less polymer matrix strength, and the bubbles tended to merge. For samples saturated by 14–18 MPa CO_2_, as mentioned in Section 3.2.2, the effect of CO_2_ plasticization on the vulcanization reaction was not significant; the main reason would be lower viscoelasticity caused by CO_2_ plasticization.

### 3.3. Effect of Vulcanization during Bubble Nucleation and Growth

#### 3.3.1. Effect of Foaming Temperature on Cell Morphology

**Figure 9:**
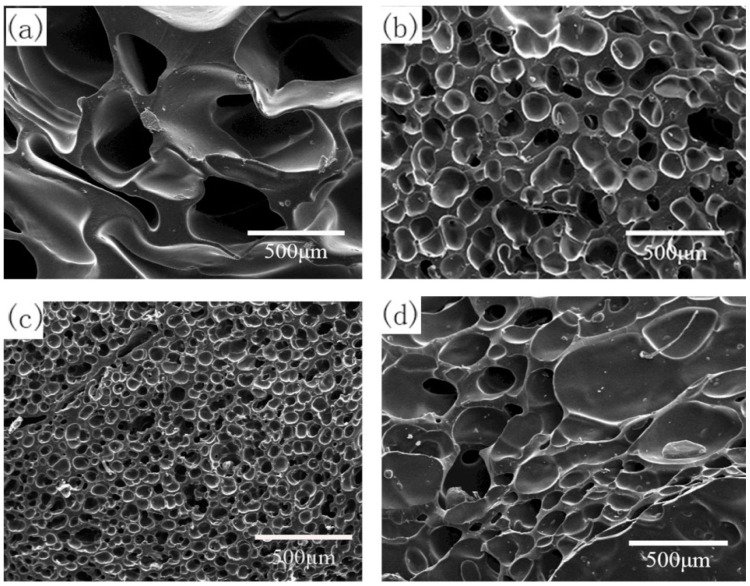
Cell morphology of foamed samples at different foaming temperatures: (**a**) 150 °C; (**b**) 160 °C; (**c**) 170 °C; (**d**) 180 °C.

The samples were saturated in 10 MPa CO_2_ for 120 min at 35 °C and then were heated to 150–180 °C for 60 min of foaming. The vulcanization degree of the samples was measured at the end of the foaming stage. The cell morphology of the obtained silicone rubber foam samples is presented in Figure 9 and Table 5. At a foaming temperature of 150 °C, the vulcanization degree after foaming was only 67.3%, and the polymer matrix strength during bubble growth was insufficient to maintain the bubbles. When the foaming temperature was 160 °C, the vulcanization degree after foaming reached 70.2%, and only a small amount of cells collapsed. When the foaming temperature was up to 170 °C, the vulcanization reaction rate increased, the polymer matrix strength was sufficient to avoid cell coalescence, and small uniform closed spherical cells were generated [32]. When the foaming temperature continued to 180 °C, vulcanization was complete and generated high crosslinking networks which limited bubble nucleation and growth. By the way, CO_2_ diffusion is faster at higher temperature, and more gas would escape out of the polymer matrix; therefore, the expansion ratio decreased.

**Figure 10:**
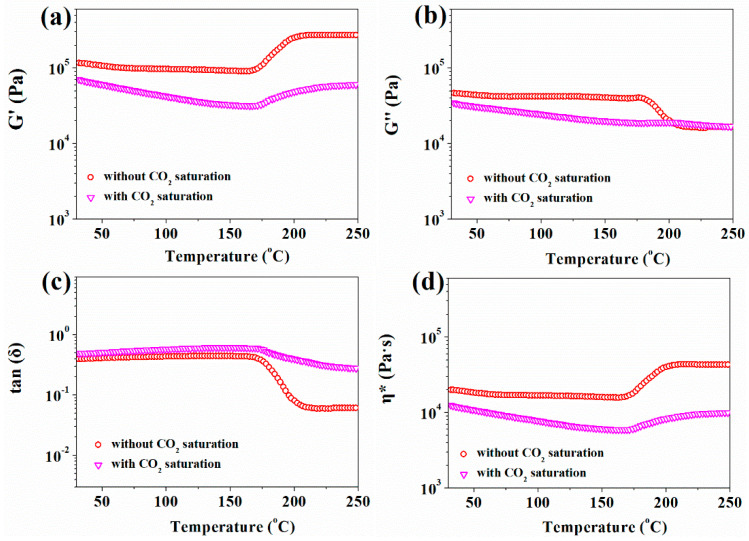
Temperature dependence of rheological properties of pre-vulcanized silicone rubber samples: (**a**) Storage modulus *G′*; (**b**) Loss modulus *G″*; (**c**) Loss angle tan (*δ*); (**d**) Complex viscosity η*.

The temperature-dependent rheological properties of pre-vulcanized silicone rubber samples are shown in Figure 10. The occurrence of a further vulcanization reaction resulted in a sudden rising of storage modulus and complex viscosity at higher temperature, but the loss modulus only had a little increase. After CO_2_ treatment, the storage modulus, loss modulus, and complex viscosity of the vulcanized sample decreased, but their scope of change with temperature was much smaller than that of a sample without CO_2_ treatment. Figure 4 also exhibited that when the temperature was around 170 °C, the storage modulus of the pre-vulcanized silicone rubber sample after CO_2_ treatment was slightly larger than its loss modulus, and the loss factor was still close to 1, which would be good for polymer foaming [23]; this is also corresponding to the foaming results.

#### 3.3.2. Effect of Foaming Time on Cell Morphology

**Figure 11:**
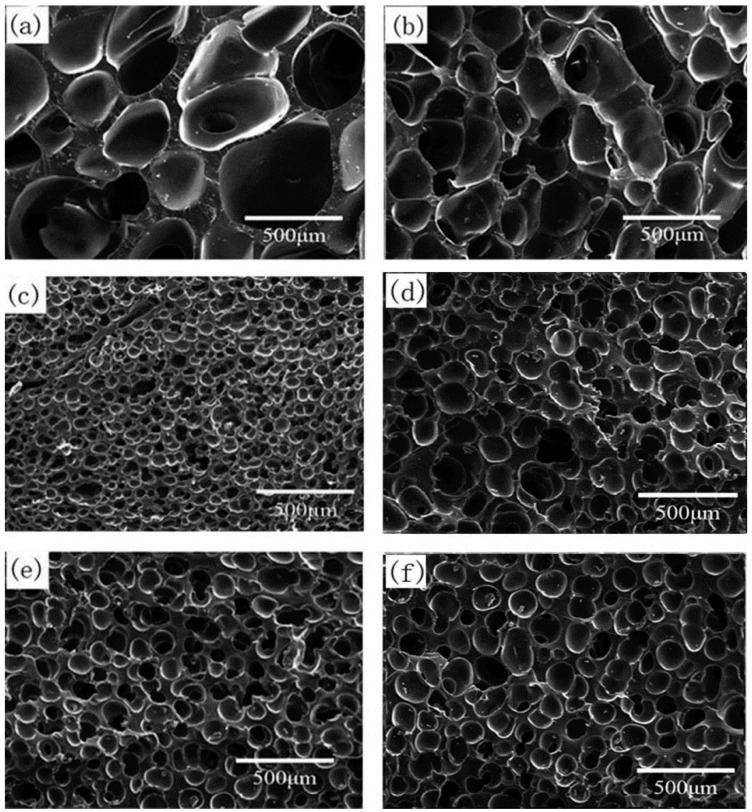
Cell morphology of foamed samples with different foaming times: (**a**) 20 min; (**b**) 40 min; (**c**) 60 min; (**d**) 80 min; (**e**) 100 min; (**f**) 120 min.

When saturated at 35 °C and 10 MPa CO_2_ for 120 min and then foamed at 170 °C for 20–120 min, the cell morphology of the foamed samples is shown in Figure 11 and Table 6. When the foaming time increased from 20 to 60 min, the vulcanization degree at the end of the foaming stage was from 74.6% to 94.4%, the cell size decreased, and the cell density increased. One reason is that the lower polymer matrix strength at the lower vulcanization degree resulted in more cell mergence; another reason is that the higher crosslinked networks at higher vulcanization degrees limited bubble growth. When the foaming time increased to 80–120 min, the cell morphologies were similar due to the complete vulcanization reaction and full cell growth.

#### 3.3.3. Effect of Temperature Rising Rate on Cell Morphology

**Figure 12:**
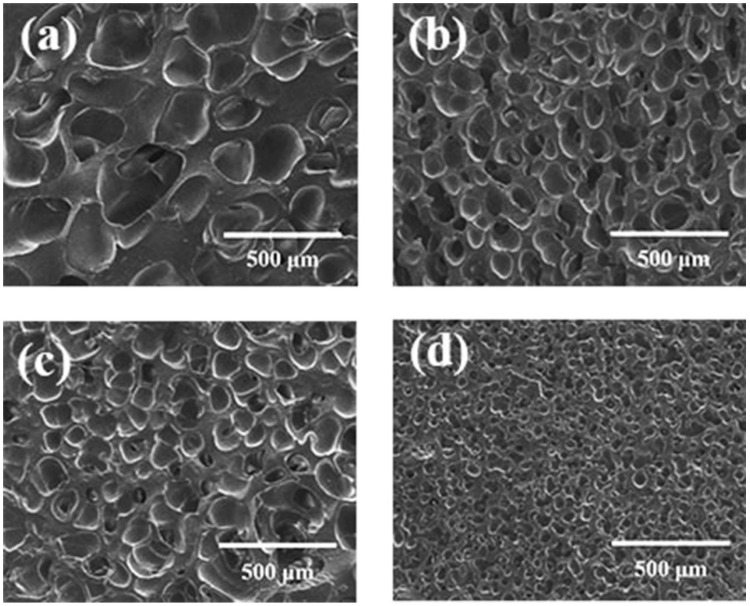
Cell morphology of foamed samples with different temperature-rising rates: (**a**) 5 °C/min; (**b**) 10 °C/min; (**c**) 15 °C/min; (**d**) 20 °C/min.

Different temperature rising rates were controlled for foaming the same CO_2_ saturated pre-vulcanized silicon rubber samples at 170 °C for 60 min. The cell morphology of obtained foams is shown in the Figure 12 and Table 7.

**Figure 13:**
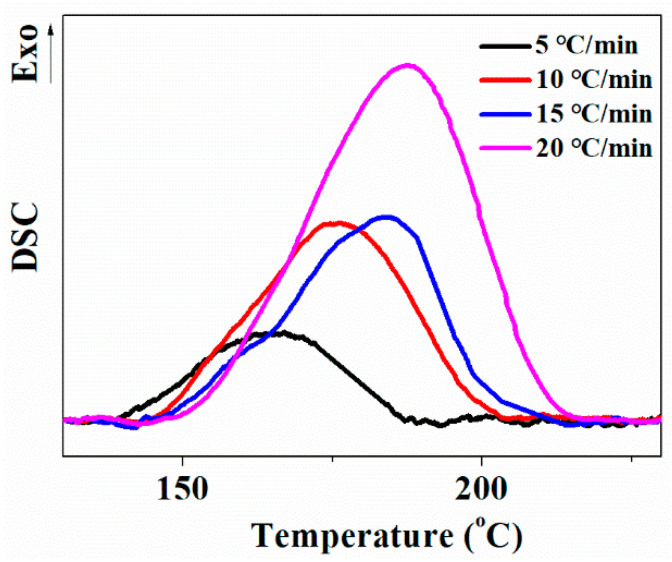
DSC curves of pre-vulcanized silicon rubber after CO_2_ treatment with different temperature-rising rates.

The higher temperature-rising rate caused a higher cell density and smaller cell size. When the temperature rising rate incresed from 5 to 20 °C/min, the expansion ratio had a little change, but the cell density increased by about two orders of magnitude, and the cell size also decreased a lot. On the one hand, a higher temperature-rising rate means more CO_2_ supersaturation in a polymer matrix, which was beneficial for bubble nucleation, and more dissolved CO_2_ would be used for bubble nucleation rather than bubble growth. On the other hand, both the vulcanization reaction rate and vulcanization degree increased during a fast temperature-rising process; as shown in Figure 13, the polymer matrix strength consequently increased rapidly, which limited bubble growth. Furthermore, bubble nucleation might also be induced by more and faster chain crosslinking.

## 4. Conclusions

The cell morphology of foamed samples could be manipulated by either vulcanization reaction or CO_2_ plasticization in the temperature rise foaming process. For the chosen methyl-vinyl silicone rubber system, there existed a pre-vulcanization degree range of 23.9–43.8% for good cell structure. During the nucleation and growth of bubbles, the effluent of dissolved CO_2_ from silicone rubber caused a delay in the initial temperature of the further vulcanization reactions, but the vulcanization reaction was still accelerated by the plasticization of dissolved CO_2_. Therefore, the lower viscoelasticity caused by good CO_2_ plasticization would be the dominating factor for appearance of bigger cells at longer CO_2_ saturation time or higher CO_2_ saturation pressure. When the foaming temperature was lower or the foaming time was shorter, less vulcanization led to a lower polymer matrix strength, and more cell mergence would happen. With increase in temperature, the elastic modulus and complex viscosity had a sudden increase because of a further vulcanization reaction. In particular, the loss factor of the vulcanized silicone rubber after CO_2_ treatment was close to 1 around 170 °C, which was beneficial for polymer foaming. Due to more bubble nucleation and faster vulcanization reaction rate, silicone rubber foam had higher cell density and smaller cell size at a higher temperature rising rate.

## Figures and Tables

**Table 1:** Parameters of foamed samples with different pre-vulcanization times.

Pre-Vulcanization Time (min)	Pre-Vulcanization Degree (%)	CO_2_ Content (g CO_2_/g SR)	Foam Density (g·cm^−3^)	Average Cell Diameter (μm)	Cell Density (×10^5^ Cells·cm^−3^)	Expansion Ratio
0	0	-	0.43 ± 0.04	465.2 ± 93.0	0.1	2.4
10	12.7	-	0.41 ± 0.02	307.2 ± 61.4	0.3	2.5
20	23.9	0.086	0.45 ± 0.03	157.7 ± 35.1	2.2	2.3
30	36.0	0.070	0.51 ± 0.03	51.6 ± 8.6	72.1	2.0
40	43.8	0.063	0.40 ± 0.04	115.0 ± 23.0	7.9	2.5
50	50.7	0.015	1.03 ± 0.05	-	-	-
60	56.6	0.008	1.05 ± 0.05	-	-	-

**Table 2:** Rheological parameters of pre-vulcanized silicone rubber samples being treated with different CO_2_ saturation times.

CO_2_ Saturation Time	c	λ	η_0_	R^2^
0 min	0.76	83.34	2,608,843.0	0.9999
30 min	0.76	80.96	2,598,144.4	0.9999
60 min	0.81	75.18	2,557,879.8	0.9999
90 min	0.71	61.03	1,272,419.6	0.9994
120 min	0.72	59.53	1,128,299.6	0.9999
150 min	0.73	32.90	618,824.5	0.9995
180 min	0.67	26.08	474,267.6	0.9993

**Table 3:** Parameters of foamed samples with different CO_2_ saturation times.

Solution Time (Min)	CO_2_ Content (g CO_2_/g SR)	Foam Density (g·cm^−3^)	Average Cell Diameter (μm)	Cell Density (×10^5^ Cells·cm^−3^)	Expansion Ratio
30	0.010	0.88 ± 0.06	-	-	1.2
60	0.025	0.76 ± 0.05	-	-	1.4
90	0.058	0.48 ± 0.03	68.7 ± 13.7	13.5	2.1
120	0.070	0.51 ± 0.03	51.6 ± 8.6	72.1	2.0
150	0.071	0.48 ± 0.03	75.6 ± 12.6	10.2	2.1
180	0.072	0.41 ± 0.01	214.0 ± 35.7	2.1	2.5

**Table 4:** Parameters of foamed samples at different CO_2_ saturation pressures.

*P* (MPa)	CO_2_ Content (g CO_2_/g SR)	Foam Density (g·cm^−3^)	Average Cell Diameter (μm)	Cell Density (×10^5^ Cells·cm^−3^)	Expansion Ratio
8	0.064	0.40 ± 0.03	102.6 ± 20.5	10.4	2.6
10	0.070	0.51 ± 0.03	51.6 ± 8.6	72.1	2.0
12	0.075	0.43 ± 0.02	94.5 ± 15.8	14.4	2.4
14	0.081	0.40 ± 0.03	124.7 ± 18.6	7.2	2.6
16	0.087	0.37 ± 0.02	132.9 ± 20.8	7.1	2.8
18	0.091	0.31 ± 0.01	201.2 ± 40.2	4.0	3.3

**Table 5:** Parameters of foamed samples at different foaming temperatures.

Temperature (°C)	Vulcanization Degree (%)	Foam Density (g·cm^−3^)	Average Cell Diameter (μm)	Cell Density (×10^5^ Cells·cm^−3^)	Expansion Ratio
150	67.3	0.46 ± 0.03	-	-	2.2
160	70.2	0.45 ± 0.02	120.9 ± 15.1	7.5	2.3
170	94.4	0.51 ± 0.03	51.6 ± 8.6	72.1	2.0
180	100.0	0.63 ± 0.03	152.0 ± 31.6	1.8	1.6

**Table 6:** Parameters of foamed samples with different foaming times.

Time (Min)	Vulcanization Degree (%)	Foam Density (g·cm^−3^)	Average Cell Diameter (μm)	Cell Density (×10^5^ Cells·cm^−3^)	Expansion Ratio
20	74.6	0.43 ± 0.01	324.4 ± 54.1	0.6	2.4
40	86.9	0.47 ± 0.03	202.2 ± 33.7	2.2	2.2
60	94.4	0.51 ± 0.03	51.6 ± 8.6	72.1	2.0
80	100.0	0.53 ± 0.02	98.3 ± 16.4	11.5	1.9
100	100.0	0.52 ± 0.02	97.2 ± 15.8	11.4	2.0
120	100.0	0.53 ± 0.03	101.6 ± 16.9	11.3	1.9

**Table 7:** Parameters of foamed samples with different temperature-rising rates.

Temperature Rising Rate (°C/min)	Foam Density (g·cm^−3^)	Average Cell Diameter (μm)	Cell Density (×10^5^ Cells·cm^−3^)	Expansion Ratio
5	0.62 ± 0.03	182.0 ± 36.4	1.6	1.6
10	0.56 ± 0.02	90.7 ± 18.1	10.7	1.8
15	0.52 ± 0.03	78.0 ± 15.6	19.1	2.0
20	0.57 ± 0.03	37.5 ± 7.5	109.1	1.8

## Data Availability

The data presented in this study are available on request from the corresponding author.
